# Adverse events in different administration routes of amiodarone: a pharmacovigilance study based on the FDA adverse event reporting system

**DOI:** 10.3389/fphar.2025.1517616

**Published:** 2025-01-27

**Authors:** Jingrong Yang, Mengfan You, Jingxin Wang, Rongfei Sun, Lili Han, Xiaonan Liu, Kaibin Niu, Kaidi Xing, Juanping Sun, Wenge Su, Yifei Wang

**Affiliations:** ^1^ Shandong University of Traditional Chinese Medicine, Jinan, China; ^2^ Affiliated Hospital of Shandong University of Traditional Chinese Medicine, Jinan, China; ^3^ Heart Center, Shandong Public Health Clinical Center, Jinan, China; ^4^ Department of Cardiology, Zhangdian District Hospital of Traditional Chinese Medicine, Zibo, China; ^5^ Department of Cardiovascular, Affiliated Hospital of Shandong University of Traditional Chinese Medicine, Jinan, China

**Keywords:** amiodarone, oral, intravenous, FDA, adverse events

## Abstract

**Background:**

Arrhythmias are prevalent cardiac disorders with significant impacts on patient quality of life and mortality. Amiodarone, a class III antiarrhythmic agent, is widely used to manage both atrial and ventricular arrhythmias due to its efficacy in prolonging the cardiac action potential and its multiple antiarrhythmic properties. While clinical trials have highlighted the safety and efficacy of amiodarone, there is limited real-world data on adverse events (AEs) associated with different administration routes. This study aims to address this gap by utilizing the U.S. Food and Drug Administration’s Adverse Event Reporting System (FAERS) to investigate the spectrum and timing of AEs related to amiodarone administration through disproportionality analysis and stratification methods.

**Methods:**

Data from the FAERS database were analyzed using disproportionality analysis and reporting odds ratio (ROR) methods for comparative analysis, and the Weibull distribution for time-to-adverse-event analysis. The study examined data from 2004 through the first quarter of 2024 to analyze adverse event signals and the time of occurrence between intravenous and oral amiodarone administration.

**Results:**

A total of 16,749 records of adverse reactions associated with amiodarone were identified. Among these, 2,412 events were related to intravenous amiodarone, and 8,220 events were related to oral amiodarone. The analysis revealed that cardiac and hepatic AEs were more common with intravenous administration, while pulmonary and thyroid-related AEs were more frequent with oral administration. Furthermore, the onset of adverse reactions varied significantly between the routes. The Weibull distribution analysis showed a median onset time of 5 days for intravenous administration compared to 74 days for oral administration. Both routes exhibited early failure-type signals, indicating a decreasing risk of AEs over time.

**Conclusion:**

Amiodarone exhibits varying adverse drug reactions and onset times across different routes of administration. Clinicians should carefully consider these differences when selecting the administration route to balance the risks of adverse reactions with therapeutic benefits.

## 1 Introduction

Arrhythmias are among the most frequently encountered cardiac disorders in clinical practice, often leading to significant complications, including death. Atrial fibrillation (AF), in particular, stands out as one of the most prevalent and clinically significant types of arrhythmia (Lévy, 2021). The clinical relevance of AF extends beyond its association with an elevated risk of systemic embolism and poor prognosis; it also markedly diminishes patients’ quality of life (QOL) (Wybraniec et al., 2023). As a result, early and comprehensive rhythm control strategies are critical in managing AF. These strategies encompass pharmacological cardioversion (PC), electrical cardioversion (EC), and pulmonary vein isolation, all of which have been shown to improve patient outcomes (Wybraniec et al., 2023). Guidelines from the European Society of Cardiology (ESC) recommend PC or EC as first-line treatment options for patients with new-onset symptomatic AF, provided there are no contraindications (Hindricks et al., 2021). Pulmonary vein isolation is a critical step in catheter ablation for treating AF. The AdmIRE Pivotal Trial demonstrated that catheter ablation significantly improves patient quality of life, reduces healthcare utilization in multiple measures, and has a favorable safety profile (Reddy et al., 2024). Pharmacological cardioversion, which utilizes antiarrhythmic drugs (AADs) administered either orally or intravenously, aims to terminate AF episodes, enhance quality of life, reduce recurrent hospitalizations, and lower overall healthcare costs (Airaksinen, 2022). VAs, such as ventricular tachycardia (VT) and ventricular fibrillation (VF), are major contributors to sudden cardiac death (SCD), which poses a critical and life-threatening risk while significantly impacting patients’ quality of life (Al-Khatib et al., 2018; Ortiz et al., 2017). Although implantable cardiac defibrillators (ICDs) are considered the gold standard for preventing SCD, effectively mitigating the risk of sudden death and extending survival, timely intervention in patients with VAs remains crucial to further reduce the likelihood of SCD (Bragg et al., 2024). The PARTITA trial revealed that, following the first appropriate electrical shock in patients with an implantable cardioverter defibrillator (ICD), ablation therapy for ventricular tachycardia (VT) significantly reduced both the risk of death and the incidence of hospitalization for heart failure (Della Bella et al., 2022). Concurrently, antiarrhythmic drugs (AADs) continue to be a cornerstone in the long-term management of recurrent VAs (AlTurki et al., 2019; Santangeli et al., 2016).

Amiodarone, a derivative of juxtafuran, is widely used in the treatment of both atrial and VAs (Hamilton et al., 2020). In the Vaughan Williams classification system, amiodarone is classified as a class III antiarrhythmic agent due to its predominant effect of potassium channel blockade, which prolongs the cardiac action potential duration (Vaughan, 1975). Additionally, amiodarone exhibits multiple antiarrhythmic properties, including sodium channel inhibition (class I), noncompetitive beta-blockade (class II), and calcium channel blockade (class IV) during phase 0 of the cardiac action potential (Ikeda et al., 1984). The oral formulation of amiodarone (200 mg/tablet) received U.S. Food and Drug Administration (FDA) approval in 1985 for treating adults with life-threatening VAs when alternative therapies are ineffective or intolerable; its intravenous formulation was approved in 1995 for the same indications (Van Herendael and Dorian, 2010). According to the 2024 guidelines developed by the European Society of Cardiology (ESC) in conjunction with the European Association for Cardiothoracic Surgery (EACTS), intravenous amiodarone is recommended for cardioversion in patients with AF accompanied by severe left ventricular hypertrophy, heart failure with reduced ejection fraction (HFrEF), or coronary artery disease in emergent situations (Van Gelder et al., 2024). Oral amiodarone therapy is recommended for patients requiring long-term use of antiarrhythmic drugs to prevent recurrence and progression of AF, especially those at high risk for AF, and should be closely monitored for possible extracardiac toxic effects (Hindricks et al., 2021; Joglar et al., 2024; DeSouza et al., 2024). In guidelines published by the European Society of Cardiology (ESC) in 2022, intravenous procainamide or amiodarone is recommended for patients with acute, hemodynamically stable ventricular tachycardia (VT) of unknown origin (Zeppenfeld et al., 2022). However, it is noted that amiodarone is associated with a higher likelihood of adverse effects compared to procainamide (Ortiz et al., 2017). Oral amiodarone also plays an important role in the management of VA as an adjunctive treatment. Overall, intravenous amiodarone is primarily used in acute situations requiring immediate intervention, while oral administration is usually used for long-term maintenance therapy. Both the EMIAT and CAMIAT trials demonstrate that amiodarone reduces the incidence of VF or arrhythmic death in patients with myocardial infarction (Cairns et al., 1997; Julian et al., 1997; Larson et al., 2022).

Despite its proven efficacy in managing VAs, amiodarone is associated with a notable incidence of adverse drug reactions (ADRs), significant extracardiac toxicity, and multiple drug-drug interactions, raising concerns about its clinical application (Apte and Kalra, 2023; Feduska et al., 2021). In clinical practice, amiodarone can be administered either intravenously or orally. While both routes are generally considered safe and effective, they may differ in the type and frequency of adverse events (AEs) experienced by patients. Current knowledge regarding AEs associated with different amiodarone administration routes primarily stems from clinical trials, with a notable lack of real-world data. Due to the stringent inclusion criteria and limited sample sizes of clinical trials, these studies may not fully capture the drug’s effects and safety across diverse populations. Consequently, the true spectrum of AEs related to various amiodarone administration routes may be broader than currently recognized. To address this gap, targeted studies are essential to evaluate the safety of amiodarone administered via different routes. The U.S. Food and Drug Administration’s Adverse Event Reporting System (FAERS) is a publicly accessible database that aggregates reports of AEs and medication errors submitted to the FDA (Yao et al., 2020). Utilizing the FAERS database, this study aims to comprehensively investigate the potential AEs associated with different routes of amiodarone administration and their timing through disproportionality analysis and stratification methods (Montastruc et al., 2011). A thorough understanding of the AEs linked to various amiodarone administration routes is crucial for optimizing clinical practice and ensuring patient safety.

## 2 Methods

### 2.1 Data sources

This study employed the FAERS database to conduct a comparative analysis of AEs associated with different routes of amiodarone administration, utilizing both discriminant and stratified analytical methods. The FAERS database serves as a global reporting system for adverse drug events, providing a vital resource for regulators and researchers to monitor and evaluate the safety of medications in real-world settings (Liu et al., 2024; Zhao et al., 2023). For our analysis, we extracted comprehensive data on amiodarone from the FAERS database, covering the period from 2004 to the first quarter of 2024. This dataset includes critical information such as demographic details, drug-specific information, reports of AEs, patient outcomes, sources of the reports, drug therapy specifics, and indications for use. This extensive data will enable a thorough investigation into amiodarone-related AEs and contribute to a detailed assessment of the drug’s safety profile.

### 2.2 Data extraction

**FIGURE 1:**
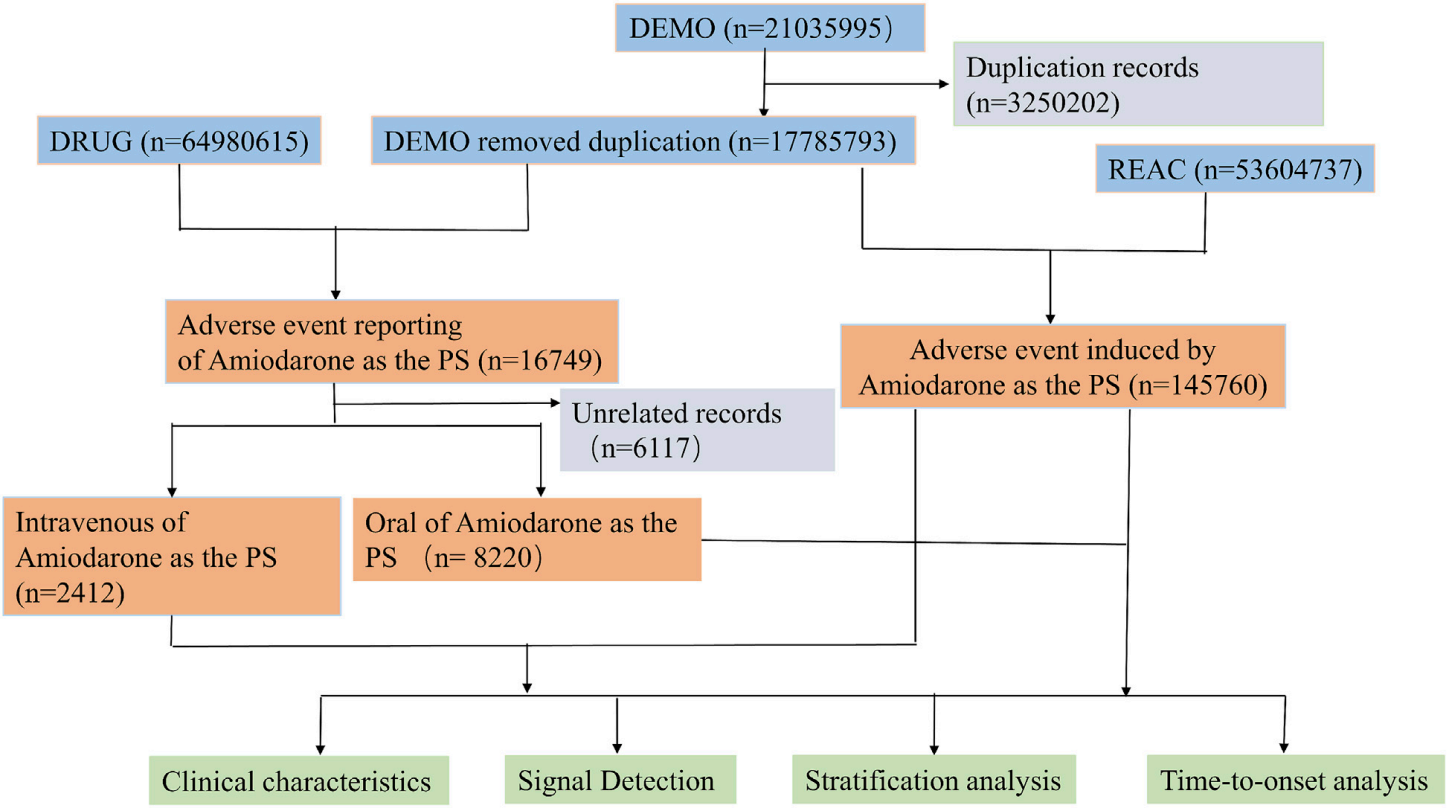
Flow chart of the study.

For the data analysis of the FAERS database, we followed the FDA-recommended procedures for duplicate removal and compiled a total of 17, 785, 793 records of personal information. Following drug-specific screening, we identified 16,749 adverse event reports related to amiodarone. Subsequent screening excluded cases with incomplete records or ambiguous routes of administration. This process led to the identification of 2,412 AEs associated with intravenous amiodarone and 8,220 AEs associated with oral amiodarone. The detailed process of screening amiodarone data from the FAERS is depicted in Figure 1, which presents a flowchart outlining the multi-stage steps of data acquisition, processing, and analysis.

## 3 Results

### 3.1 Descriptive analysis

From 2004 through the first quarter of 2024, we collected 2,412 adverse reaction events associated with intravenous amiodarone administration and 8,220 events associated with oral amiodarone administration from the FAERS database. In terms of outcomes, there were 3,675 outcomes recorded for intravenous administration and 12,048 for oral administration. We will conduct an in-depth analysis of these data to explore the specific impact of different administration routes on patient safety. The distribution of basic clinical features is detailed in Table 1. We used the Pearson chi-square test to assess statistical relevance and set the level of statistical significance at a two-tailed threshold of p < 0.05. AEs associated with intravenous drug use were notably more frequent among males, accounting for 54.9%, compared to females at 37%. In terms of body weight, it was more concentrated in the 50–100 kg group (20.8%). Age-wise, there was a significant clustering in the 65–85 years age bracket, representing 45.9% of the total, followed by the 18–64.9 years age group with 29.1%. Regarding the country of the reporter, the United States had a high concentration at 39.3%, with France trailing at 16.5%. In terms of outcomes, other outcomes were most prevalent at 35.2%, with hospitalization outcomes close behind at 32.7%.

**TABLE 1:** Clinical characteristics of patients with adverse events under different administration routes of Amiodarone.

Characteristics	Intravenous of amiodarone	Oral of amiodarone	Statistics^a^	p value^b^
X	Overall	Overall		
	(N = 2,412)	(N = 8,220)		
Sex, n (%)				
F	893 (37.0%)	3,044 (37.0%)	0.00	0.99
M	1,325 (54.9%)	4,713 (57.3%)	4.39	0.04
Missing	194 (8.0%)	463 (5.6%)		
WT (kg), n (%)				
<50	70 (2.9%)	121 (1.5%)	21.62	<0.001
>100	105 (4.4%)	353 (4.3%)	0.02	0.90
50∼100	501 (20.8%)	2,242 (27.3%)	41.21	<0.001
Missing	1736 (72.0%)	5,504 (67.0%)		
Age (year), n (%)				
<18	72 (3.0%)	62 (0.8%)	74.57	<0.001
>85	132 (5.5%)	861 (10.5%)	55.10	<0.001
18∼64.9	702 (29.1%)	1,227 (14.9%)	252.38	<0.001
65∼85	1,106 (45.9%)	4,306 (52.4%)	31.82	<0.001
Missing	400 (16.6%)	1764 (21.5%)		
Country, n (%)				
US	947 (39.3%)	2,632 (32%)	43.80	<0.001
FR	397 (16.5%)	2018 (24.6%)	69.53	<0.001
IT	145 (6.0%)	732 (8.9%)	20.63	<0.001
CA	129 (5.3%)	226 (2.7%)	30.02	<0.001
DE	99 (4.1%)	320 (3.9%)	0.22	0.64
Others	595 (24.7%)	1988 (24.2%)	0.24	0.63
Missing	100 (4.1%)	304 (3.7%)		
Reported, n (%)				
CN	136 (5.6%)	1,349 (16.4%)	180.10	<0.001
HP	307 (12.7%)	391 (4.8%)	193.17	<0.001
LW	2 (0.1%)	418 (5.1%)	122.98	<0.001
MD	728 (30.2%)	2,247 (27.3%)	7.50	0.006
OT	688 (28.5%)	1775 (21.6%)	50.32	<0.001
PH	397 (16.5%)	1,589 (19.3%)	10.12	0.001
RN	7 (0.3%)	2 (0.0%)	12.60	<0.001
Missing	147 (6.1%)	449 (5.5%)		
Reporting year, n				
2004	41	179	2.10	0.15
2005	88	203	9.74	0.002
2006	58	146	3.91	0.048
2007	60	160	2.70	0.10
2008	68	156	7.68	0.006
2009	38	127	0.01	0.92
2010	40	157	0.65	0.42
2011	34	123	0.10	0.76
2012	85	186	11.94	<0.001
2013	118	190	44.15	<0.001
2014	90	379	3.42	0.06
2015	119	461	1.65	0.20
2016	129	569	7.53	0.006
2017	127	644	18.30	<0.001
2018	245	1,184	28.90	<0.001
2019	250	1,250	36.08	<0.001
2020	231	732	1.02	0.31
2021	163	546	0.04	0.84
2022	166	439	8.26	0.004
2023	209	306	98.83	<0.001
2024	53	83		
Outcome, n (%)				
X	Overall	Overall		
	(N = 3,675)	(N = 12,048)		
CA	1 (0.0%)	2 (0.0%)	0.00	1
DE	364 (9.9%)	1,215 (10.1%)	0.10	0.75
DS	76 (2.1%)	496 (4.1%)	33.72	<0.001
HO	1,200 (32.7%)	4,533 (37.6%)	30.04	<0.001
LT	519 (14.1%)	1,090 (9.0%)	78.96	<0.001
OT	1,293 (35.2%)	4,072 (33.8%)	2.41	0.12
RI	55 (1.5%)	192 (1.6%)	0.17	0.68
Missing	167 (4.5%)	448 (3.7%)		

Note: Data in bold indicates statistical significance.

^a^ The Pearson chi-square test, chi-squared value (χ2).

^b^ P value, two-tailed threshold of p < 0.05.

For oral medications, AEs were also more concentrated among males at 57.3% compared to females at 37%. In terms of body weight, it was also more concentrated in the 50–100 kg group (27.3%). In terms of age distribution, the 65–85 years age group had the highest concentration at 52.4%, followed by the 18–64.9 years age group with 14.9%. The United States led in terms of the country of the reporter with 32%, with France at 24.6%. When it came to outcomes, hospitalization outcomes were most common at 37.6%, followed by other outcomes at 33.8%.

It is important to note significant differences in AEs between the two routes of administration in the following demographics: males, individuals weighing less than 50 kg, and those in the 50–100 kg weight range. Additionally, significant differences were observed across all age groups. In terms of outcomes, there were notable disparities between the two pathways for disability, hospitalization, and life-threatening outcomes.

### 3.2 Signal intensity of adverse reactions for different routes of administration

To investigate whether there are differences in AEs between the two routes of administration, we utilized the Reporting Odds Ratio (ROR) as our assessment tool. Table 2 shows the two-by-two contingency table associated with it. Positive signals were defined by an ROR value of at least 3 and a lower limit of the 95% Confidence Interval (CI) greater than 1. Based on these criteria, we screened for positive signals in each administration route, ranked them according to the ascending order of the ROR 95% CI values, and selected the top 15 AEs for further analysis. These findings are summarized and presented in Table 3.

**TABLE 2:** The two-by-two contingency table.

3.2 linked tables in chapters			
	Target adverse drug event	Other adverse drug events	Sums
Amiodarone	a	b	a + b
Other drugs	c	d	c + d
Sums	a + c	b + d	a + b + c + d

3.3 Linked Tables in Chapters			
	Target adverse drug event with amiodarone	Other adverse drug events with amiodarone	Sums
Oral	a	b	a + b
Intravenous	c	d	c + d
Sums	a + c	b + d	a + b + c + d

**TABLE 3:** Comparative analysis of AE signal intensity across administration methods.

	PT	a	ROR	ROR (95% Cl)
Intravenous	Compensatory sweating	3	1104.94	1104.94 (319.82–3817.37)
	Electrocardiogram	4	850.04	850.04 (296.61–2436.12)
	T wave alternans femoral	5	552.58	552.58 (220.32–1385.95)
	Hernia incarcerated lymphatic fistula	5	445.63	445.63 (179.12–1108.66)
	Junctional ectopic tachycardia	4	442.02	442.02 (159.61–1224.14)
	Infusion site phlebitis	14	399.14	399.14 (231.96–686.83)
	Cardiac arrest neonatal	6	281	281 (123.7–638.34)
	Appendicolith	13	258.61	258.61 (148.24–451.18)
	Infusion site necrosis	5	215.85	215.85 (88.31–527.59)
	Arrhythmic storm	5	167.45	167.45 (68.77–407.74)
	Myocardial stunning	3	162.49	162.49 (51.54–512.3)
	Gastrointestinal dysplasia	3	154.9	154.9 (49.17–487.99)
	Decompensated hypothyroidism	9	148.52	148.52 (76.58–288.02)
	Optic disc haemorrhage	4	142.59	142.59 (52.84–384.8)
	Gammopathy	4	127.02	127.02 (47.13–342.31)
Oral	Mitochondrial aspartate aminotransferase increased	8	3962.88	3962.88 (1051.27–14938.42)
	Iodine uptake abnormal	6	2971.99	2971.99 (743.24–11884.09)
	Iodine overload	4	371.48	371.48 (124.18–1111.22)
	Eosinophilia myalgia syndrome	3	212.27	212.27 (63.31–711.68)
	Deposit eye	30	208.46	208.46 (142.23–305.51)
	Electrocardiogram RR interval prolonged	4	160.64	160.64 (57.25–450.71)
	Femoral hernia incarcerated	5	148.6	148.6 (59.25–372.64)
	Pulmonary toxicity	445	129.14	129.14 (117.16–142.35)
	Lymphatic fistula	5	119.83	119.83 (48.18–298.08)
	Bone marrow granuloma	4	104.27	104.27 (37.83–287.4)
	Laryngeal haematoma	4	102.48	102.48 (37.2–282.29)
	Secondary hyperthyroidism	5	99.06	99.06 (40.06–244.96)
	Scrotal haematocoele	5	97.76	97.76 (39.55–241.65)
	Corneal deposits	39	96.84	96.84 (70.03–133.9)
	Decompensated hypothyroidism	18	82.08	82.08 (51.06–131.94)

Note: PT, preferred term; a, number of cases with available; ROR, reporting odds ratio.

The signal intensity for the following adverse reactions was more pronounced with the intravenous route of administration: compensatory sweating (n = 3, ROR = 1,104.94), electrocardiogram t wave alternans (n = 4, ROR = 850.04), femoral hernia incarcerated (n = 5, ROR = 552.58), lymphatic fistula (n = 5, ROR = 445.63), junctional ectopic tachycardia (n = 4, ROR = 442.02), infusion site phlebitis (n = 14, ROR = 399.14), cardiac arrest neonatal (n = 6, ROR = 281), appendicolith (n = 13, ROR = 258.61), infusion site necrosis (n = 5, ROR = 215.85), arrhythmic storm (n = 5, ROR = 167.45), myocardial stunning (n = 3, ROR = 162.49), gastrointestinal dysplasia (n = 3, ROR = 154.9), decompensated hypothyroidism (n = 9, ROR = 148.52), optic disc hemorrhage (n = 4, ROR = 142.59), and gammopathy (n = 4, ROR = 127.02). Among them, we identified 10 new AEs not listed in the product insert, including compensatory sweating, incarcerated femoral hernia, lymphatic fistula, neonatal cardiac arrest, appendicolith, infusion site necrosis, myocardial stunning, gastrointestinal dysplasia, optic disc hemorrhage, and gammopathy.

The signal strength for the following adverse reactions was notably higher with the oral route of administration: mitochondrial aspartate aminotransferase increased (n = 8, ROR = 3,962.88), abnormal iodine uptake (n = 6, ROR = 2,971.99), iodine overload (n = 4, ROR = 371.48), eosinophilia-myalgia syndrome (n = 3, ROR = 212.27), eye deposits (n = 30, ROR = 208.46), prolonged electrocardiogram RR interval (n = 4, ROR = 160.64), incarcerated femoral hernia (n = 5, ROR = 148.6), pulmonary toxicity (n = 445, ROR = 129.14), lymphatic fistula (n = 5, ROR = 119.83), bone marrow granuloma (n = 4, ROR = 104.27), laryngeal hematoma (n = 4, ROR = 102.48), secondary hyperthyroidism (n = 5, ROR = 99.06), scrotal hematocele (n = 5, ROR = 97.76), corneal deposits (n = 39, ROR = 96.84), and decompensated hypothyroidism (n = 18, ROR = 82.08). We also identified six new AEs not mentioned in the specification for the oral route, including eosinophilia myalgia syndrome, incarcerated femoral hernia, lymphatic fistula, bone marrow granuloma, laryngeal hematoma, and scrotal hematocoele.

Our comparative analysis revealed that intravenous administration is more likely to trigger cardiac-related AEs than oral administration, including T wave alternans, junctional ectopic tachycardia, neonatal cardiac arrest, arrhythmic storm, and myocardial stunning. Intravenous administration is also more likely to result in infusion site phlebitis and necrosis. Notably, intravenous administration is associated with a higher likelihood of AEs not included in the product insert.

In contrast, oral administration is less likely to cause adverse reactions not mentioned in the product insert. AEs related to the respiratory system, iodine, and thyroid functions are more common with oral administration and include abnormal iodine uptake, iodine overload, pulmonary toxicity, laryngeal hematoma, secondary hyperthyroidism, and decompensated hypothyroidism.

### 3.3 Distributional characteristics of common adverse reactions

**FIGURE 2:**
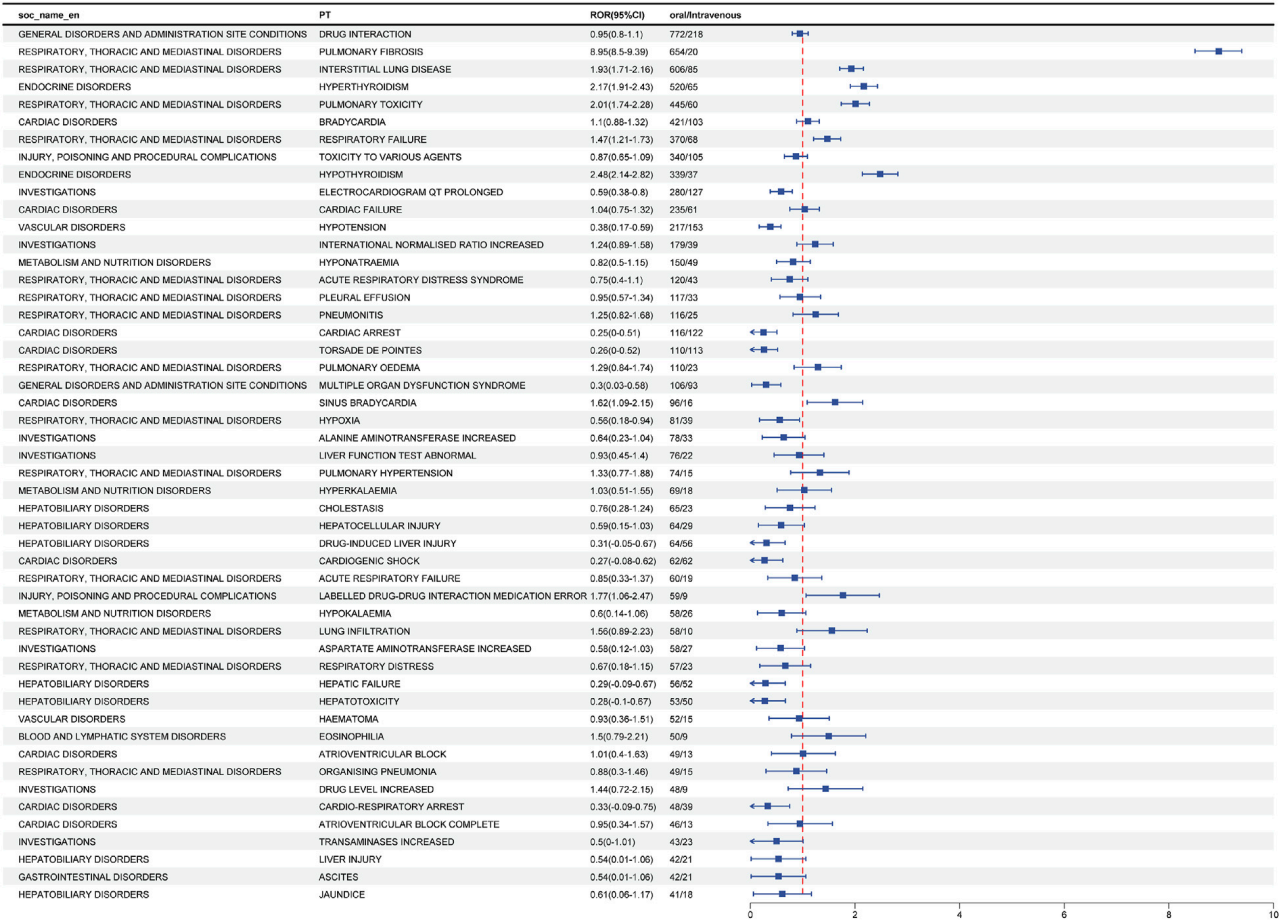
Analysis of differential risk signals for different administration routes of Amiodarone. Odds ratios (ROR) for the top 50 AEs are reported in the figure, 95% CI. PT, preferred term; SOC, system organ classes; ROR, reporting odds ratio.

To study whether common adverse reactions caused by amiodarone are more likely to occur in the intravenous or oral route of administration. We conducted a ratio of reports (ROR) analysis (a technique used in multivariate analysis) for the study. Table 2 shows the two-by-two contingency table associated with it. We sorted the data based on the number of adverse reaction events, selecting the top 50 adverse reactions and categorizing them by their system organ class. The results are illustrated in Figure 2.

Positive signals for adverse reactions were identified by comparing the ROR and its confidence intervals. Adverse reactions were considered more likely with oral administration if the ROR was greater than 1 and the confidence interval did not include 1. Based on this criterion, the following adverse effects were found to be more prevalent with oral administration: pulmonary fibrosis, interstitial lung disease, hyperthyroidism, pulmonary toxicity, respiratory failure, hypothyroidism, sinus bradycardia, and drug-drug interaction medication errors.

Conversely, adverse reactions were more likely to occur with intravenous administration if the ROR was less than 1 and the confidence interval did not include 1. The adverse reactions more frequently associated with intravenous administration included prolonged QT interval on ECG, hypotension, cardiac arrest, torsade de pointes, multiple organ dysfunction syndrome, hypoxia, drug-induced liver injury, cardiogenic shock, hepatic failure, hepatotoxicity, and cardio-respiratory arrest.

In summary, different routes of administration are associated with specific risks of adverse reactions. Oral administration may increase the risk of certain pulmonary and thyroid-related adverse effects, while intravenous administration is linked to a higher risk of cardiac and hepatic adverse reactions.

### 3.4 Induction time of relevant adverse reactions under different routes of administration

**FIGURE 3:**
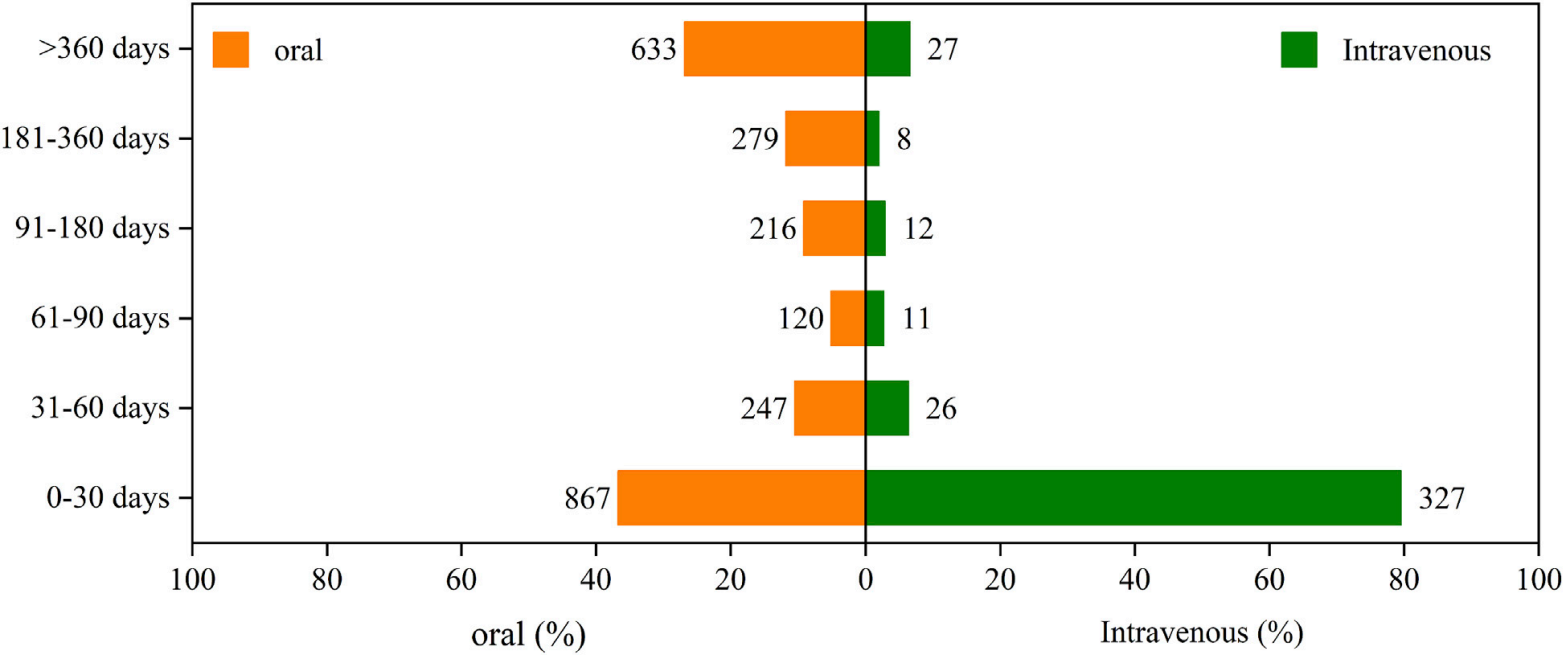
The induction time of adverse reactions associated with different routes of administration.

By analyzing temporal data on adverse reactions to amiodarone from the FAERS, we constructed Figure 3 to illustrate the incidence of AEs by different routes of administration. Our analysis revealed that the incidence of AEs in the first month was significantly higher for intravenous administration (79.56%) compared to oral administration (36.71%).

Specifically, during subsequent time periods, the incidence rates for oral administration were higher than those for intravenous administration. For the periods of 31–60 days, 61–90 days, 91–180 days, 181–360 days, and over 360 days, the incidence rates for oral administration were 10.46%, 5.08%, 9.14%, 11.81%, and 26.8%, respectively. In comparison, the rates for intravenous administration were 6.33%, 2.68%, 2.92%, 1.95%, and 6.57% for the same periods.

Overall, both oral and intravenous administration routes showed a gradual decrease in the incidence of AEs over time. This decline may be attributed to patient adaptation to the drug or other factors influencing the treatment course.

### 3.5 Time-to-onset analysis

Table 4 presents the results of the onset time and weighted signal proportion (WSP) analyses for clinically preferred signals of amiodarone AEs associated with both intravenous and oral administration. The median time to onset of AEs was 5 days (interquartile range [IQR]: 2–23.5 days) for intravenous administration, compared to 74 days (IQR: 14–366 days) for oral administration. Notably, the shape parameter β and the upper limit of its 95% confidence interval (CI) for both administration routes in the WSP analysis were less than 1. This indicates that the clinical preference signals for both intravenous and oral administration tend to exhibit early failure, suggesting a gradual decrease in the risk of AEs over time.

**TABLE 4:** The analysis of the onset time of priority signals for intravenous injection and oral administration.

Prioritization				Weilbull distribution				Failure type
	Case	TTO (days)		Scale parameter		Shape parameter		
	n	Median (IQR)	Min-max	α	95% CI	β	95% CI	
Intravenous	411	5 (2–23.5)	1–7,341	23.21	17.67–28.75	0.43	0.40–0.46	Early failure
Oral	2,362	74 (14–366)	1–9,435	196.45	180.46–212.43	0.52	0.51–0.54	Early failure

Note: n, number of cases with available time-to-onset; IQR, interquartile range; TTO, Time-to-onset.

## 4 Disscusion

Through an in-depth analysis of data from the FDA Adverse Event Reporting System (FAERS), this study aimed to investigate how different routes of administration affect various aspects of amiodarone-related AEs. Demographic data indicates that adverse reactions from both routes of administration are predominantly concentrated among males and individuals with a body weight of 50–100 kg, particularly in the age group of 65–85 years. This aligns with studies showing that the risk of AEs, such as pulmonary toxicity, increases with age (Ernawati et al., 2008), and older patients are more susceptible to adverse pulmonary events (Jackevicius et al., 2011). Notably, there are significant differences in adverse reactions between the two administration routes among certain populations: males, those weighing less than 50 kg, and those within the 50–100 kg weight range. Additionally, significant differences are observed across all age groups. In individuals under the age of 65, intravenous administration has been linked to a higher incidence of adverse reactions, while in those aged 65 and older, oral administration appears to lead to a greater number of adverse reactions. This observation does not rule out the possibility that individuals over 65 may be affected by the long-term use of oral medication for the control of chronic diseases. The outcome analysis revealed that a greater proportion of life-threatening outcomes were associated with intravenous administration, whereas a higher percentage of disability and hospitalization outcomes were linked to oral administration.

Upon reviewing the signal intensity of adverse reactions for the two administration routes, we determined that lung- and thyroid-related adverse reactions exhibited higher signal intensity with oral administration, while heart- and infusion site-related adverse reactions showed higher signal intensity with intravenous administration. Additionally, when analyzing the distribution characteristics of the more frequently occurring adverse reactions, we found that lung- and thyroid-related adverse reactions were more prevalent in the oral administration route, whereas cardiac and hepatic adverse reactions were more commonly observed in the intravenous administration route.

These findings align with prior research and further validate the existence of differences in AEs associated with the two distinct modes of amiodarone administration. Adverse reactions at the administration site, such as infusion site phlebitis and infusion site necrosis, are unique and distinct AEs associated with intravenous administration. Research has revealed that there are variations in the incidence of cardiac-related adverse effects depending on the route of administration. For instance, Shenthar et al. and Noedkind et al. found that intravenous administration is more likely to result in torsade de pointes (Nordkin et al., 2021; Shenthar et al., 2017). Lévy’s study highlighted that intravenous amiodarone can lead to hypotension, potentially due to polysorbate 80, a solvent used in the formulation (Lévy, 2021).

Although some studies have indicated that intravenous administration rarely leads to QT interval prolongation (Dias et al., 2002; Mujović et al., 2020; Kodama et al., 1997; Gelman et al., 2024), while other studies have shown that oral administration is associated with QT interval prolongation (Mujović et al., 2020; Kodama et al., 1997; Plomp et al., 1985), our findings suggest that QT interval prolongation is more likely to occur with intravenous administration. Junctional ectopic tachycardia (JET) is a common arrhythmia observed after surgery for congenital heart disease (Rochelson et al., 2022). While intravenous amiodarone has been demonstrated to reduce the incidence of postoperative junctional ectopic tachycardia in children and is considered a first-line treatment (Rochelson et al., 2022; Sasikumar et al., 2021; Arvind et al., 2021), our study found that intravenous amiodarone is more likely to lead to adverse effects such as ectopic tachycardia and neonatal cardiac arrest, which remain significant concerns in pediatric applications.

Regarding liver-related adverse reactions, pharmacologic liver injury, liver failure, and hepatotoxicity are predominantly observed with intravenous administration. Several case reports highlight hepatic adverse reactions associated with intravenous administration, such as Mohamed et al., who reported acute hepatic failure induced by intravenous amiodarone (Mohamed et al., 2020), and Liwag et al., who reported hepatotoxicity occurring within 24 h of intravenous amiodarone administration (Liwag et al., 2022). Although transient asymptomatic elevations in serum aminotransferase levels occur in about 25% of patients taking oral amiodarone, complications such as symptomatic hepatitis, cirrhosis, and liver failure are relatively uncommon (Biancatelli et al., 2019). Additionally, Lahbabi et al. published a case report of acute hepatitis during amiodarone infusion, but liver function tests returned to normal following the discontinuation of intravenous administration and continuation of oral amiodarone (Lahbabi et al., 2012). This finding aligns with our study, which also found that liver-related adverse reactions are more likely with intravenous administration. This may be related to the rapid increase in serum amiodarone concentration due to intravenous administration, though the hepatotoxicity of the solvent polysorbate 80 cannot be excluded (Sileno et al., 2020).

Among pulmonary adverse reactions, such as pulmonary fibrosis, interstitial lung disease, pulmonary toxicity, and respiratory failure, these are predominantly associated with oral administration of amiodarone. Feduska et al. demonstrated that pulmonary toxicity from amiodarone is mainly observed following long-term oral therapy, while it is relatively rare with short-term intravenous administration (Feduska et al., 2021). This difference may be attributed to the pharmacokinetics of amiodarone, which has a notably longer half-life of 13–142 days for oral administration compared to 18–36 h for intravenous administration. The extended half-life associated with the oral route facilitates the prolonged accumulation of amiodarone and its metabolite N-desethylamiodarone (DEA) in the lungs, which contributes to the development of pulmonary adverse effects (Feduska et al., 2021).

Regarding endocrine system adverse reactions, secondary hyperthyroidism and decompensated hypothyroidism are more frequently associated with oral administration. This finding aligns with previous literature. Thyroid dysfunctions, including amiodarone-induced thyrotoxicosis (AIT) and hypothyroidism (AIH), have been reported in 14%–18% of patients on long-term oral amiodarone (Medić et al., 2022; Martino et al., 2001). Given that the iodine content of amiodarone is approximately 37% by weight, a daily dose of 200–600 mg exceeds the recommended daily iodine intake (150 μg) by a factor of 35–140, resulting in excessive iodine consumption (Medić et al., 2022).

Additionally, intravenous administration tends to accelerate the onset of adverse effects compared to oral administration. This may be due to the fact that intravenous administration allows the drug to rapidly enter the circulatory system, thereby expediting its effects.

Antiarrhythmic therapy is a key component of a comprehensive treatment strategy for heart failure (HF). Patients with heart failure often experience arrhythmias that can exacerbate HF symptoms or diminish the effectiveness of treatment (Zhang et al., 2018). Therefore, effective control of arrhythmias is crucial for improving the prognosis of patients with heart failure. Despite the significant efficacy of amiodarone in antiarrhythmic therapy, its numerous adverse effects may limit its use in the treatment of heart failure. In this context, other medications or non-pharmacological therapies may be another option.

According to the research by Scara et al. (2024), non-pharmacological treatments for heart failure, including lifestyle modifications, physical activity, and electrotherapy, have been shown to be both efficacious and safe. Structured exercise training, in particular, has demonstrated effectiveness in improving functional status, enhancing quality of life, and reducing mortality risk in patients with heart failure, especially in those with reduced ejection fraction. Additionally, electrical therapies such as catheter ablation and cardiac resynchronization therapy (CRT) have been proven effective in improving cardiac function and alleviating heart failure symptoms. For patients who do not meet the criteria for CRT or do not respond to it, cardiac contractility modulation (CCM), an emerging therapy, offers new treatment options. These non-pharmacological therapies provide a more diverse array of treatment options for heart failure patients, contributing to optimized outcomes and improved quality of life.

### 4.1 Limitations

Despite the clear advantages of leveraging real-world data mining strategies based on the FAERS database for research, it is important to acknowledge the inherent limitations of all pharmacovigilance databases, including FAERS. Firstly, the FAERS database relies on voluntary reporting, which introduces risks of reporting bias and underreporting. Consequently, issues such as false reports, incomplete data, inaccuracies, and delays in reporting can affect the comprehensiveness and reliability of adverse reaction data. Secondly, while adverse event reporting systems can identify statistical associations between drug use and AEs, they do not establish causality. Therefore, although these systems can highlight potential adverse effects associated with drug use, they cannot confirm whether these effects are directly caused by the drug itself. It is also challenging to exclude the potential influence of other underlying conditions or concomitant medications on the occurrence of AEs.Thirdly, the data in the FAERS database are often derived from reports related to specific patient populations and may reflect only particular time periods. This limitation necessitates further validation through additional studies to generalize findings to broader populations. Lastly, our study focused on the impact of different routes of administration on AEs but did not account for other potential confounders, such as medication timing, dosage, and patient adherence. To confirm the observed associations, extensive experimental studies, clinical trials, case-control studies, and cohort studies are required.

### 4.2 Clinical significance

In our pharmacovigilance study, we conducted a comprehensive analysis of a substantial dataset of real-world safety information to investigate the AEs associated with amiodarone when administered via intravenous versus oral routes. Our study specifically examined variations in adverse event proportions across different age groups and identified that the route of administration may influence the type of AEs observed. We found that oral administration was more frequently associated with pulmonary and thyroid-related AEs, whereas intravenous administration was more commonly linked to cardiac, systemic, and hepatic adverse reactions. Consequently, oral administration may be preferable for mitigating cardiac and hepatic adverse effects compared to intravenous administration.

Our analysis using the Weibull distribution revealed that the median onset of action for intravenous administration was significantly shorter at 5 days compared to 74 days for oral administration. Additionally, the Weighted Signal Probability (WSP) analysis indicated that adverse event signals for amiodarone, regardless of the administration route, exhibited an early failure type pattern—where the risk of AEs diminished over time. These findings have significant implications for clinical practice and pharmacovigilance, offering healthcare professionals valuable insights for assessing the risks associated with different administration routes of amiodarone. This, in turn, can aid in devising safer and more effective therapeutic regimens for patients.

## 5 Conclusion

In summary, the AEs and their timing associated with amiodarone vary depending on the route of administration. Despite its extensive use for treating atrial and VAs due to its potent antiarrhythmic properties, clinicians must thoroughly understand the potential side effects linked to different dosage forms when selecting the appropriate administration route. A comprehensive understanding of these variations allows for the optimization of amiodarone therapy, potentially reducing adverse effects and enhancing therapeutic outcomes.

## Data Availability

The original contributions presented in the study are included in the article/supplementary material, further inquiries can be directed to the corresponding authors.
